# Practical application of Westgard Sigma rules with run size in analytical biochemistry processes in clinical settings

**DOI:** 10.1002/jcla.23665

**Published:** 2020-12-03

**Authors:** SongQing Peng, JinFei Zhang, WuQiong Zhou, WeiLin Mao, Zhong Han

**Affiliations:** ^1^ Department of Clinical Laboratory Shengzhou People's Hospital Shengzhou Branch of the First Affiliated Hospital of Zhejiang University Shengzhou China; ^2^ Key laboratory of digestive system diseases of Shengzhou Shengzhou People’s Hospital Shengzhou China

**Keywords:** quality control, run size, six sigma, Westgard rule

## Abstract

**Background:**

The performance of 18 routine chemical detection methods was evaluated by the sigma (*σ*) metric, and Westgard Sigma rules with run size were used to establish internal quality control (IQC) standards to reduce patient risks.

**Materials and methods:**

External quality assessment (EQA) and internal quality control data from 18 assays in a biochemical laboratory were collected from January to June 2020. The sigma values of each assay were calculated, based on the bias, total error allowable, and coefficient of variation, appropriate quality control rules were selected. According to the quality goal index, the main causes of poor performance were determined to guide quality improvement.

**Results:**

At IQC material level 1, seven of the 18 assays achieved five sigma (excellent), and five assays (UA, Crea, AMY, TC and Na) showed world‐class performance. At IQC material level 2, 14 of the 18 assays achieved 5 sigma (excellent), and thirteen assays (UA, ALT, CK, Crea, AMY, K, AST, ALP, Na, LDH, Mg, TC and GGT) showed world‐class performance. The quality goal index (QGI) was calculated for items with analysis performance <5 sigma, and the main causes of poor performance were determined to guide quality improvement.

**Conclusions:**

Westgard sigma rules with run size are an effective tool for evaluating the performance of biochemical assays. These rules can be used to more simply and intuitively select the quality control strategy of related items and reduce the risk to patients.

## 1 INTRODUCTION

The Clinical and Laboratory Standards Institute (CLSI) C24‐ED4 guidelines^1^ recommend the implementation of risk‐based statistical quality control (SQC). It is recommended to design a limited interval SQC for continuous analysis processes, that is, to implement a quality control event before and after testing a limited group of samples, and the number of samples in this group is the run size, the number of quality control (QC) events in the continuous analytical process is the QC frequency. However, many SQC design tools fail to clearly provide the SQC frequency selection and design parameters required for continuous analysis process. Westgard took MaxE (NUF) = 1 as the target to determine run size and established Westgard sigma rules with the run size.^2^

Six Sigma is a technology that can improve the quality process management of enterprises, and it was first applied at Motorola. The purpose was to meet the quality requirements of ‘zero defects’. Six Sigma indicates the international quality level. A six sigma analysis means that 99.99966% of the results are error free, corresponding to 3.4 defects for every million opportunities.^3^ Since the application of six sigma quality management in laboratory medicine in 2000, more and more laboratories in China have begun to apply the six sigma management method in the quality evaluation of detection systems. So far, the sigma methodology has mainly been applied in the evaluation of immunoassay and biochemical tests.^4^, ^5^

In this study, Westgard sigma rules with a run size management method were used to evaluate the detection performance of clinical test items in a biochemical laboratory with the objectives of improving the quality level of the clinical biochemical laboratory and reducing the risk to patients.

## 2 MATERIALS AND METHODS

### 2.1 Analyser and assays

A Hitachi 7600 analysis system was used to perform 18 routine biochemical assays: total cholesterol (TC), alanine aminotransferase (ALT), gamma‐glutamyl transferase (GGT), amylase (AMY), creatine kinase (CK), aspartate aminotransferase (AST), lactate dehydrogenase (LDH), alkaline phosphatase (ALP), glucose (Glu), urease (Urea), creatinine (Crea), uric acid (UA), total protein (TP), calcium (Ca), magnesium (Mg), chloride (Cl), sodium (Na) and potassium (K).

ALT, AST, GGT, ALP, CK, Urea, UA, Crea, LDH, AMY, Glu, TC and TP were tested with reagents obtained from Meikang, Mg from Woko, Ca from Diasys, and K, Na and Cl from Hitachi. Calibration was performed with reagents from Roche, and IQC products were obtained from Beckman Coulter (lot 1: M901101, lot 2: M901103). All EQA samples were provided by the National Center for Clinical Laboratories (lots: VE002, VE005, 202001, 202002, 202011, 202012).

### 2.2 Statistical analysis

The precision is expressed by the coefficient of variation (CV) and calculated by the following formula: CV (%) = [Standard Deviation/Mean] × 100.^6^ The internal quality control data were collected between January and June 2020 at our clinical biochemical laboratory.

The average value of the absolute percentage differences was used to evaluate bias in our laboratory (Table 1) and was calculated based on EQA samples.

**TABLE 1:** Sigma, bias, quality goal index (QGI), total allowable error (TEa), and coefficient of variation (CV) values of the two levels of quality control for the assays

Parameter	Average bias (%)	TEa	Level 1			Level 2		
			CV	Sigma value	QGI	CV	Sigma value	QGI
ALT	1.78	16	3.13	4.55	0.38	1.18	12.03	
AST	3.13	15	2.48	4.80	0.84	1.19	9.97	
GGT	7.08	11	1.52	2.59	3.11	0.65	6.07	
LDH	0.88	11	2.11	4.80	0.28	1.34	7.58	
CK	3.97	15	2.48	4.44	1.07	1.03	10.74	
AMY	7.58	15	1.06	7.03		0.72	10.29	
ALP	5.54	18	2.31	5.40		1.33	9.39	
Glu	0.55	7	1.30	4.97	0.28	1.22	5.29	
Urea	0.59	8	1.81	4.10	0.22	1.54	4.82	0.26
Crea	0.22	12	1.55	7.62		1.11	10.66	
UA	0.31	12	1.08	10.81		0.76	15.35	
TP	1.03	5	0.93	4.28	0.74	0.91	4.39	0.76
Na	0.50	4	0.52	6.77		0.38	9.13	
K	0.32	6	1.01	5.65		0.57	9.99	
Ca	1.34	5	1.27	2.88	0.70	1.06	3.46	0.84
Mg	3.55	15	2.84	4.04	0.83	1.60	7.18	
Cl	2.97	4	1.06	0.98	1.87	0.86	1.21	2.30
TC	0.07	9	1.32	6.79		1.27	7.03	

Abbreviations: ALP, alkaline phosphatase; ALT, alanine aminotransferase; AMY, amylase; AST, aspartate aminotransferase; Ca, calcium; CK, creatine kinase; Cl, chloride; Crea, creatinine; GGT, gamma‐glutamyl transferase; Glu, glucose; K, potassium; LDH, lactate dehydrogenase; Mg, magnesium; Na, sodium; TC, total cholesterol; TP, total protein; UA, uric acid; Urea, urease.

Total allowable error (TEa) includes systematic error and random error. The recommended allowable error values for assays according to the requirements of the Health Industry Standards of the People's Republic of China (WS/T 403‐2012) are presented in Table 1.

Calculate the sigma metric using the following formula^7^:

$$\text{Sigma}\;\text{metric}=\frac{\left(\text{TEa}‐|\text{Bias}|\right)}{\text{CV}}$$

The QGI was calculated using the standard equation: QGI = Bias∕(1.5 × CV%). This indicator can help to identify the main reasons for the lower sigma level in the test performance of clinical chemistry projects and may help to select the best quality improvement plan.^8^, ^9^, ^10^ The quality goal index (QGI) was calculated for assays with analysis performance <5 sigma, and the main causes of poor performance were determined to guide quality improvement. When QGI > 1.2, improvement of the accuracy should be prioritized; when 0.8 ≤ QGI ≤ 1.2, both accuracy and precision of the analyte should be improved; When QGI < 0.8, the precision of the analyte needs to be improved.

## 3 RESULTS

The performances and sigma values of the 18 assays in the Hitachi 7600 analysis system were calculated in our laboratory, and the results are shown in Tables 1 and 2, intuitively assess the performance of the analyte at each quality control material level. According to the sigma level, the assay performances were divided into six grades, namely unacceptable (*σ* < 2), poor (2 ≤ *σ* < 3), marginal (3 ≤ *σ* < 4), good (4 ≤ *σ* < 5), excellent (5 ≤ *σ* < 6) and world class (*σ* ≥ 6).

**TABLE 2:** IQC procedures selected for 18 assays

Parameter	Level 1	Level 2	IQC procedure
	Sigma value	Sigma value	
ALT	4.55	12.03	1_3s_/2_2s_/R_4s_/4_1s_ with N4 and R200 (Level 1)^a^; 1_3s_ with N2 and R1000 (Level 2)
AST	4.80	9.97	1_3s_/2_2s_/R_4s_/4_1s_ with N4 and R200 (Level 1)^a^; 1_3s_ with N2 and R1000 (Level 2)
GGT	2.59	6.07	1_3s_/2_2s_/R_4s_/4_1s_/8_x_ with N4 and R45 (Level 1)^a^; 1_3s_ with N2 and R1000 (Level 2)
LDH	4.80	7.58	1_3s_/2_2s_/R_4s_/4_1s_ with N4 and R200 (Level 1)^a^; 1_3s_ with N2 and R1000 (Level 2)
CK	4.44	10.74	1_3s_/2_2s_/R_4s_/4_1s_ with N4 and R200 (Level 1)^a^; 1_3s_ with N2 and R1000 (Level 2)
AMY	7.03	10.29	1_3s_ with N2 and R1000
ALP	5.40	9.39	1_3s_/2_2s_/R_4s_ with N2 and R450 (Level 1)^a^; 1_3s_ with N2 and R1000 (Level 2)
Glu	4.97	5.29	1_3s_/2_2s_/R_4s_/4_1s_ with N4 and R200 (Level 1)^a^; 1_3s_/2_2s_/R_4s_ with N2 and R450 (Level 2)
Urea	4.10	4.82	1_3s_/2_2s_/R_4s_/4_1s_ with N4 and R200
Crea	7.62	10.66	1_3s_ with N2 and R1000
UA	10.81	15.35	1_3s_ with N2 and R1000
TP	4.28	4.39	1_3s_/2_2s_/R_4s_/4_1s_ with N4 and R200
Na	6.77	9.13	1_3s_ with N2 and R1000
K	5.65	9.99	1_3s_/2_2s_/R_4s_ with N2 and R450 (Level 1)^a^; 1_3s_ with N2 and R1000 (Level 2)
Ca	2.88	3.46	1_3s_/2_2s_/R_4s_/4_1s_/8_x_ with N4 and R45
Mg	4.04	7.18	1_3s_/2_2s_/R_4s_/4_1s_ with N4 and R200 (Level 1)^a^; 1_3s_ with N2 and R1000 (Level 2)
Cl	0.98	1.21	1_3s_/2_2s_/R_4s_/4_1s_/8_x_ with N4 and R45
TC	6.79	7.03	1_3s_ with N2 and R1000

Note

R, run size of patient samples between QC events, R45 represents a run size of 45 patient samples between QC events, and similar definitions apply to R1000, R450, and R200.

N, total number of control measurements per run, N2 represents two measurements at a single control material level or one measurement at two control material levels, and a similar definition applies to N4.

Abbreviations: ALP, alkaline phosphatase; ALT, alanine aminotransferase; AMY, amylase; AST, aspartate aminotransferase; Ca, calcium; CK, creatine kinase; Cl, chloride; Crea, creatinine; GGT, gamma‐glutamyl transferase; Glu, glucose; IQC, Internal quality control; K, potassium; LDH, lactate dehydrogenase; Mg, magnesium; Na, sodium; TC, total cholesterol; TP, total protein; UA, uric acid; Urea, urease.

^a^ IQC procedure selected in this study.

At IQC material level 1, seven of the 18 assays achieved 5 sigma (excellent), and five assays (UA, Crea, AMY, TC and Na) showed world‐class performance (Table 1). At IQC material level 2, 14 of the 18 assays achieved 5 sigma (excellent), and thirteen assays (UA, ALT, CK, Crea, AMY, K, AST, ALP, Na, LDH, Mg, TC and GGT) showed world‐class performance (Table 1).

The IQC procedures for the 18 assays at different IQC material levels are detailed in Table 2. For example, for the level 1 IQC, five assays showed a performance of ≥6 sigma level‐one control rule, 1_3s_, with one control measurement at two IQC material levels (N2) per IQC event and a run size of 1000 patient samples between IQC events (R1000). Three assays (GGT, Cl and Ca) showed a performance of less than 4 sigma, full multi‐rules, namely 1_3s_/2_2s_/R_4s_/4_1s_/8_x_ with N4 and R45, were needed for IQC. For level 2 IQC, thirteen assays achieved 6 sigma, and two assays (Cl and Ca) a performance of less than 4 sigma (Table 2). Data show that the Sigma method can scientifically optimize each IQC material level.

The quality goal index (QGI) was calculated for items with analysis performance <5 sigma, and the main causes of poor performance were determined to guide quality improvement. Six assays (Urea, LDH, Glu, ALT, Ca and TP) exhibited precision problems at one or more IQC levels; four assays (Mg, AST, CK and Ca) showed that there were problems of accuracy and precision at one or more IQC levels; two assays (Cl and GGT) showed low accuracy problems at one or more IQC levels (Table 3).

**TABLE 3:** QGI and quality improvement measures of the 18 routine biochemistry assays

QGI	Parameters		Problem
	Level 1	Level 2	
QGI < 0.8	Urea LDH Glu ALT Ca TP	Urea TP	Precision
0.8 ≤ QGI ≤ 1.2	Mg AST CK	Ca	Precision and accuracy
QGI > 1.2	Cl GGT	Cl	accuracy

Abbreviations: ALT, alanine aminotransferase; AST, aspartate aminotransferase; Ca, calcium; CK, creatine kinase; Cl, chloride; GGT, gamma‐glutamyl transferase; Glu, glucose; LDH, lactate dehydrogenase; Mg, magnesium; QGI, Quality goal index; TP, total protein; Urea, urease.

## 4 DISCUSSION

Sigma metrics have many uses in the clinical laboratory: The method helps laboratories select appropriate quality control rules, and the number of quality control measurements per batch, When installing a new analytical system, it confirms the quality of the method and establishes quality improvement programs.^11^, ^12^, ^13^

CLSI C24‐ED4 guidelines propose statistical quality control (SQC) based on patient risk, IQC changes from monitoring the stability of the detection system to reducing the risk of injury to patients with unreliable results. The risk‐based SQC design needs to determine three elements: number of quality control products, quality control rules and sample run size.

Westgard Sigma rules with run size are new tool for laboratory quality control strategy, proposed by Westward at the annual meeting of American Association for Clinical Chemistry (AACC) in 2018. Compared with the original Westgard sigma rules, it increases the run size under different sigma levels. At the same time, some quality control rules and the quantity of each quality control measurement were adjusted. It can be more convenient to help the laboratory to choose the appropriate quality control strategy when testing samples are batched in large quantities, the reasonable run size is helpful to detect the deviation in time, minimize the number of unacceptable patient outcomes affected by systematic deviation and achieve quality control objectives based on patient risk.

The sigma level of some of the assays, investigated in this study, show the differences between different research groups.^14^, ^15^, ^16^, ^17^ There were several main reasons for this phenomenon: the first was source selection of the TEa target; the second was the difference between the algorithms used to evaluate CV and bias; the third was that the different types of reagents, analysers and IQC materials were used. In addition, there is a difference in the sigma metrics between the two IQC levels in this study (Tables 1 and 2), and this fact is not specific for this study, as it has been found in other studies.^6^, ^18^, ^19^, ^20^ For example, Zhou et al^6^ found that the determination of ALT in the level 1, run size is 45, but run size is 200 in level 2. Therefore, QC frequency maybe on level 1 is four times more than QC frequency on level 2. Our laboratory formulates QC strategy according to the low QC level of sigma metrics. For example, IQC procedure 13s/22s/R4s/41s with N4 and R200 would be selected, and run size was 200 for ALT assay (Table 2). The purpose is to effectively reduce the error of detection system and improve the accuracy of the test results.

At present, most biochemical tests in the laboratory have reached more than 4 sigma. According to the sigma metric, most of the biochemical test results are suitable. Only the analysis if Cl, Ca and Na are below 4 sigma, which may be related to reagent choice and frequent changes in reagent batch number. At present, the sample size of each batch of biochemical inspection items is below 400, and two levels of quality control materials are used once. AMY, ALP, Crea, UA, Na, K and TC detection can meet the requirements of risk management. For ALT, AST, GGT, LDH, CK, Glu, Urea, TP, Ca, Mg and Cl analysis, one or two internal quality controls should be added in the detection process to eliminate false positive or negative wrong results. The higher the QC frequency is, the easier it is to detect system errors and prevent health hazards to patients, but the higher the QC frequency is, the heavier the economic burden. Proper QC frequency is essential for timely detection of test errors that signal unstable performance.

There are still some deficiencies in the QC plan designed in this study. First, our design basically reduces patient health risks by increasing the frequency of QC. However, QC frequency is very difficult to control because different testing items have different QC frequencies. Second, personalized quality control strategies will increase labour volume, and a high QC frequency will increase the related economic burden.

## 5 CONCLUSIONS

The use of Westgard sigma rules with run size is rarely reported in domestic literature. In theory, the Westgard sigma rule with run size can also be used for quality control of other high‐throughput continuous work instrument projects, but its practicability needs further clinical verification and discussion.

## CONFLICT OF INTEREST

None of the authors have any commercial or other associations that might pose a conflict of interest.

## Data Availability

The data are available upon reasonable request.
